# Impact of blood collection tubes on thrombin generation assay results: a comparison of citrate and CTAD

**DOI:** 10.1038/s41598-026-64702-6

**Published:** 2026-07-31

**Authors:** Stefan Ulbing, Fabian Schäfer, Stefan Koch, Christoph Dibiasi, Marion Wiegele, Peter Quehenberger, Friederike Töpper, Eva Schaden, Johannes Gratz

**Affiliations:** 1 Department of Anaesthesia, Intensive Care Medicine and Pain Medicine, Clinical Division of General Anaesthesia and Intensive Care Medicine, Medical University of Vienna, Währinger Gürtel 18-20, 1090 Vienna, Austria; 2 Ludwig Boltzmann Institute Digital Health and Patient Safety, Währinger Straße 104/10, 1090 Vienna, Austria; 3 Department of Laboratory Medicine, Medical University of Vienna, Währinger Gürtel 18-20, 1090 Vienna, Austria; 4 Department of Anesthesiology and Intensive Care Medicine, Campus Benjamin Franklin, Charité – Universitätsmedizin Berlin, Hindenburgdamm 30, 12203 Berlin, Germany

**Keywords:** Coagulation, Thrombin generation assay, Citrate-theophylline-adenine-dipyridamole blood collection tubes, Biochemistry, Biomarkers, Diseases, Medical research

## Abstract

Accurate assessment of haemostasis is essential in both research and clinical practice. Thrombin generation assays (TGA) provide a comprehensive evaluation of coagulation; however, results can be influenced by preanalytical conditions, including the choice of blood collection tubes. Citrate-theophylline-adenosine-dipyridamole (CTAD) tubes are recommended by the manufacturer of the Ceveron^®^ alpha TGA system due to their ability to inhibit platelet activation. Nevertheless, CTAD tubes are costly, less widely available, and not manufactured in small volumes suitable for specific patient populations, such as paediatric cohorts. To evaluate whether standard citrate tubes can serve as a viable alternative, we prospectively compared TGA parameters from blood collected in 3.2% sodium citrate tubes versus CTAD tubes in 12 healthy volunteers. Assays were performed using low and high phospholipid micelle concentrations (RC low/RC high). Significant differences between citrate and CTAD were observed for all parameters with the RC low reagent. In contrast, only lag time showed significant differences with the RC high reagent. In conclusion, while citrate tubes may be used for TGA, the type of blood collection tube can influence measurements under specific assay conditions and should be considered a relevant factor when comparing and interpreting TGA results.

**Supplementary Information:**

The online version contains supplementary material available at 10.1038/s41598-026-64702-6.

## Introduction

Assessing and treating a patient’s coagulation state—whether procoagulant, with an increased risk of thromboembolism, or anticoagulant, with an increased risk of bleeding—remains a major clinical challenge. Laboratory evaluation of haemostasis is therefore essential for diagnosing and managing bleeding and thrombotic disorders^1^.

Beyond conventional coagulation tests, such as prothrombin time (PT) and activated partial thromboplastin time (aPTT), as well as viscoelastic assays such as rotational thromboelastometry, the thrombin generation assay (TGA) has emerged as a valuable diagnostic tool, particularly in the research setting. TGA provides a dynamic assessment of thrombin generation over time, reflecting both procoagulant and anticoagulant pathways and offering insights into a patient’s overall haemostatic capacity^2–4^.

In our research laboratory, TGA is performed using the Ceveron® alpha TGA system (Technoclone, Vienna, Austria; software version V 2.1.2.2). For blood collection, citrate-theophylline-adenosine-dipyridamole (CTAD) tubes are recommended by the manufacturer^5,6^. These tubes contain citrate, theophylline, adenosine, and dipyridamole, which chelate calcium and inhibit platelet activation, thereby reducing preanalytical variability^7–10^. CTAD also allows for prolonged storage before sample processing^10^. However, disadvantages include higher cost (currently approximately + 40% at our centre) and limited availability compared with standard trisodium citrate tubes.

As an alternative, trisodium citrate tubes are widely used for TGA^11^. Citrate prevents coagulation by binding calcium ions, which can later be reintroduced to restore coagulation activity^12^. Citrate tubes are inexpensive, readily available in hospital wards and familiar to clinical staff. Nevertheless, tube composition may influence results: in 2020, Luca et al. demonstrated that several commercially available citrate tubes from different manufacturers yielded different TGA results^13^.

During the design of an ongoing study at our institution, we encountered an additional practical challenge: CTAD tubes are not available in the same range of volumes as citrate tubes. In particular, there is a lack of low-volume tubes, complicating their use in certain patient populations—especially infants, for whom blood sampling volume is a critical consideration. These practical limitations, combined with the restricted availability and higher cost of CTAD tubes, prompted the present study. The aim of this study was to compare TGA results obtained from blood collected in 3.2% sodium citrate tubes and CTAD tubes when measuring TGA with low and high phospholipid micelle concentrations.

## Methods

### Study design and ethics

This prospective laboratory in vitro pilot study was approved by the Ethics Committee of the Medical University of Vienna (EK 1937/2022, 23/02/2023, Dr. Juergen Zezula) and conducted in accordance with the Declaration of Helsinki and Good Clinical Practice guidelines. The study was performed between February 2023 and March 2024. Written informed consent was obtained from all participants. Blood sampling and TGA measurements were carried out at the Medical University of Vienna, Department of Anaesthesia, Intensive Care and Pain Medicine, Clinical Division of General Anaesthesia and Intensive Care Medicine.

This investigation was performed in parallel with a previously published study on viscoelastic detection of blood spiked with enoxaparin und argatroban^14^. Accordingly, baseline measurements of blood counts and standard laboratory test results have been reported previously^14^. For the current study, no in vitro spiking was performed.

### Participants and preanalytical conditions

As previously described^14^, venous blood was drawn from 12 healthy volunteers using an 18G Venflon™ Pro Safety cannula (BD, USA). Blood was collected sequentially into 3.2% sodium citrate (0.109 mol/l) tubes and CTAD tubes (both Greiner Bio-One, Kremsmünster, Austria). Exclusion criteria were: (i) known or newly identified haemostatic disorders during the course of the study; (ii) use of anticoagulants and/or platelet aggregation therapy within 14 days; (iii) known renal or hepatic impairment; and (iv) concurrent participation in an interventional study at the time of screening. Within five minutes after sampling, blood was centrifuged at 4500 *g* for 15 min to obtain platelet-poor plasma (PPP) by removing the supernatant, which was stored at − 80 °C until analysis. TGA was performed in batches at a later time point.

### Laboratory measurements

TGA was performed on the fully automated Ceveron^®^ alpha system (Technoclone, Vienna, Austria; Software Version V 2.1.2.2). Our protocol for TGA measurements has been described previously^5,6,15^. Briefly, the assay was initiated by adding 15 µl of (i) RC low reagent (low concentration of phospholipid micelles) or (ii) RC high reagent (high concentration of phospholipid micelles, identical concentration of tissue factor as in RC low), 35 µl CaCl_2_ (25 mM) and 20 µl reaction buffer to 40 µl platelet-poor plasma pre-warmed to 37 °C. Thrombin activity was quantified after cleavage of a fluorogenic substrate (Z-Gly-Gly-Arg-AMC, 40 µl) by thrombin. Thrombin activity was plotted over time, yielding a curve characterized by lag time (time from initiation to first detectable formation of thrombin; tLag, min), time to maximum thrombin concentration (tPeak, min), and peak thrombin level (Peak, nM). After reaching the peak, thrombin levels decline. The velocity index (VI, nM min⁻¹) was calculated as Peak/(tPeak−tLag) while the area under the curve (AUC) represents the endogenous thrombin potential (ETP, nM). All measurements were performed in duplicate, and mean values were used for analysis. All reagents were purchased from Technoclone (Vienna, Austria).

Baseline measurements included blood cell counts and conventional coagulation assays. Prothrombin time (PT, Owren method), activated partial thromboplastin time (aPTT), thrombin time, and plasma fibrinogen levels (Clauss method) were measured using an STA R Max 2 coagulometer (Diagnostica Stago, Asnières, France). Antithrombin activity was assessed by a heparin cofactor–based thrombin inhibition assay (STA-STACHROM ATIII; Diagnostica Stago). Complete blood cell counts, including erythrocytes, leukocytes, platelets, and haemoglobin, were analysed on a Sysmex XN-1500 haematology analyser (Sysmex, Vienna, Austria).

### Statistics

Since this investigation was conducted as a pilot study, no formal sample size calculation was carried out. Instead, the sample size was defined based on our prior experience with comparable experimental studies^15,16^, as well as on previously published guidelines for determining sample sizes in pilot studies^17^. Continuous variables are reported as median (25th–75th percentile). Pearson correlation coefficients and Bland-Altman analysis were used to assess agreement between citrate and CTAD samples. A two-sided *p*-value ≤ 0.05 was considered statistically significant. All statistical analyses were conducted on an exploratory basis. Analyses and graphical representations were generated using R version 4.4.3^18^.

## Results

Twelve healthy volunteers were included. Samples from one participant were excluded from analysis as they were identified as an outlier. Bland–Altman plots including this outlier are provided in the supplementary materials. Table 1 summarizes participant characteristics and conventional coagulation assays.

**Table 1:** Participant characteristics and conventional coagulation assays.

	*N* = 11^a^	Reference range (female/male)
Age (years)	29 (27, 32)	
Sex	F: 6 (55%), M: 5 (45%)	
Weight (kg)	70 (58.5, 80)	
Height (cm)	175 (169.5, 183)	
Red blood cells (T.l^−1^)	4.5 (4.1, 5.05)	F: 3.8–5.2
		M: 4.4–5.8
Haemoglobin (g.l^−1^)	136 (124.5, 143.5)	F: 120–160
		M: 135–180
Haematocrit (%)	40.4 (36.65, 42.95)	F: 35–47
		M: 40–52
Leukocytes (G.l^−1^)	6.08 (4.53, 6.63)	4–10
Platelet count (G.l^−1^)	271 (235.5, 303)	150–350
Prothrombin time (s)	27.34 (26.05, 29.86)	24.6–32.7
International normalised ratio (–)	1.0 (1.0, 1.1)	
Activated partial thromboplastin time (s)	34.3 (33.5, 35.7)	27–41
Thrombin time (s)	15.80 (15.45, 16.15)	< 21.0
Clauss’ fibrinogen concentration (g.l^− 1^)	2.49 (2.31, 3.12)	2–4
Antithrombin activity (%)	113 (100, 115)	80–120

Data are given as median and interquartile range [IQR]. F = female, M = male, ^a^Median (first quartile, third quartile).

### TGA with RC low reagent

**Fig. 1:**
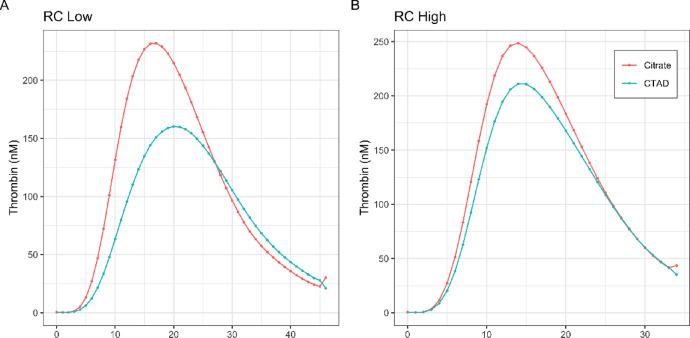
Thrombin generation curves of the two sampling groups measured with the RC low reagent and the RC high reagent. (**A**) Mean thrombin generation curves of the two sampling groups measured with the RC low reagent. (**B**) Mean thrombin generation curves of the two sampling groups measured with the RC high reagent.

**Fig. 2:**
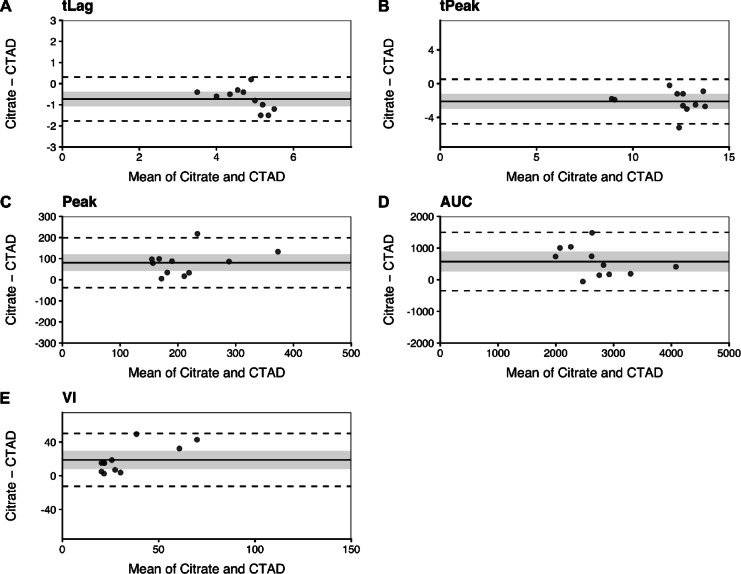
Differences between the two sampling groups in TGA with the RC low reagent in a Bland-Altman plot. (**A**) Bland–Altman plot for the tLag TGA parameter measured with the RC low reagent. (**B**) Bland–Altman plot for the tPeak TGA parameter measured with the RC low reagent. (**C**) Bland–Altman plot for the Peak TGA parameter measured with the RC low reagent. (**D**) Bland–Altman plot for the AUC TGA parameter measured with the RC low reagent. (**E**) Bland–Altman plot for the VI TGA parameter measured with the RC low reagent.

TGA parameters differed significantly between citrate and CTAD tubes (Table 2; Fig. 1A). Figure 2 presents Bland–Altman plots illustrating these differences for assays performed with the RC low reagent.

**Table 2:** TGA parameters measured with RC low reagent for citrate and CTAD group.

RC low					
	Citrate *N* = 11^a^	CTAD *N* = 11^a^	Difference^b^	95% CI^b^	*p*-value^b^
Lag time (min)	4.50 (4.10, 4.70)	4.90 (4.60, 5.90)	− 0.73	− 1.1, − 0.37	0.001
Time to peak (min)	11.70 (9.80, 12.00)	13.90 (12.00, 14.50)	− 2.1	− 3.0, − 1.2	< 0.001
Peak (nM)	219 (199, 331)	164 (118, 203)	81	40, 121	0.001
Velocity index (nM/min)	31 (28, 63)	18 (14, 28)	19	8.2, 30	0.003
Endogenous thrombin potential (nM)	2990 (2575, 3369)	2496 (1739, 2840)	574	258, 891	0.002

Data are given as a median and interquartile range [IQR]. ^a^Median (first quartile, third quartile); ^b^Paired t-test. CI = confidence interval.

### TGA with RC high reagent

**Fig. 3:**
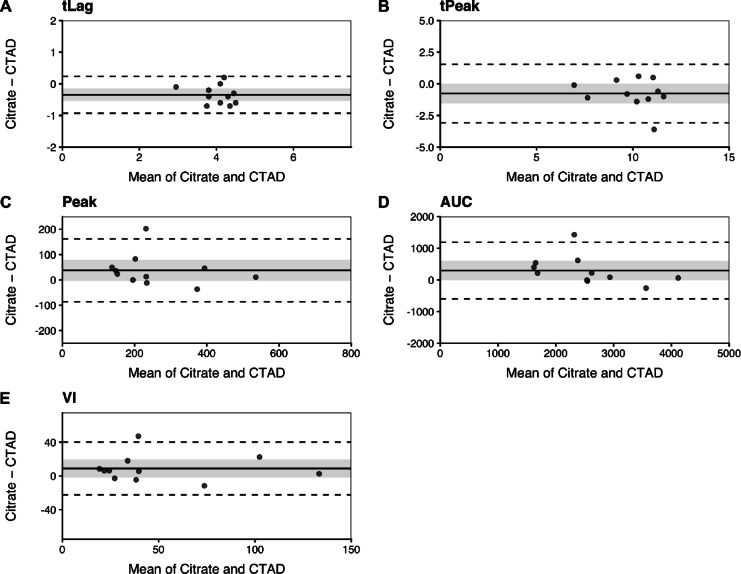
Differences between the two sampling groups in TGA with the RC high reagent in a Bland-Altmann plot. (**A**) Bland–Altman plot for the tLag TGA parameter measured with the RC high reagent. (**B**) Bland–Altman plot for the tPeak TGA parameter measured with the RC high reagent. (**C**) Bland–Altman plot for the Peak TGA parameter measured with the RC high reagent. (**D**) Bland–Altman plot for the AUC TGA parameter measured with the RC high reagent. (**E**) Bland–Altman plot for the VI TGA parameter measured with the RC high reagent.

No significant differences in TGA parameters were observed between citrate and CTAD samples, except for a difference in lag time. (Table 3; Fig. 1B). Figure 3 presents Bland–Altman plots illustrating the comparison of assays performed with the RC high reagent.

**Table 3:** TGA parameters measured with RC high reagent for citrate and CTAD group.

RC high					
	Citrate *N* = 11^a^	CTAD *N* = 11^a^	Difference^b^	95% CI^b^	*p*-value^b^
Lag time (min)	4.00 (3.60, 4.20)	4.10 (4.00, 4.60)	− 0.35	− 0.55, − 0.15	0.003
Time to peak (min)	9.50 (9.30, 11.00)	10.80 (9.00, 11.60)	− 0.76	− 1.6, 0.03	0.057
Peak (nM)	239 (166, 355)	196 (130, 371)	38	− 4.9, 80	0.077
Velocity index (nM/min)	43 (26, 68)	29 (19, 80)	8.9	− 1.8, 20	0.094
Endogenous thrombin potential (nM)	2689 (1922, 3034)	2512 (1576, 2896)	296	− 10, 603	0.057

Data are given as a median and interquartile range [IQR]. ^a^ Median (first quartile, third quartile); ^b^ Paired t-test. CI = confidence interval.

## Discussion

In this prospective in vitro pilot study, we compared thrombin generation assays performed on samples collected in CTAD versus standard citrate tubes under different assay conditions. Significant differences were observed across all parameters when using the RC low reagent, whereas with the RC high reagent, only lag time showed significant differences.This indicates that the influence of CTAD versus citrate tubes is conditional on assay phospholipid concentration.

In CTAD tubes, platelet activation is inhibited via several mechanisms. Theophylline, a xanthine derivative, acts as a phosphodiesterase inhibitor and prevents the breakdown of cAMP^19^. Adenosine binds to G protein-coupled receptors A2A and A2B, thereby increasing intracellular cAMP levels^20^. Dipyridamole increases intraplatelet cAMP concentrations by inhibiting cyclic nucleotide phosphodiesterase and blocks the uptake of adenosine^21^. Together, these mechanisms suppress platelet activation and consequently reduce the release of procoagulant mediators such as platelet factor 4^10^. The differences between RC low and RC high measurements in our findings may be explained by the greater relevance of preanalytical suppression of procoagulant mediators by CTAD when TGA is performed with low rather than high phospholipid concentrations. Phospholipids are added to TGA reagents to provide a standardized phospholipid concentration for coagulation reactions and thereby improve assay precision^22,23^. According to the manufacturer’s specifications, the RC low reagent is intended for the assessment of bleeding tendency, whereas the RC high reagent is recommended for monitoring anticoagulant therapy^24^. Our findings suggest that the RC low assay may not only be more responsive to physiological changes but also more susceptible to preanalytical influences. In addition to phospholipid concentration, previous studies have demonstrated that tissue factor concentration also affects TGA results^22,25,26^. However, because both assays in the present study used the same tissue factor concentration, the observed differences cannot be attributed to variations in tissue factor.

The implications of the differences between RC low and RC high may be even more pronounced when extrapolating our findings to platelet-rich plasma (PRP)-based TGA. Because PRP-based TGA preserves the contribution of platelets to thrombin generation, it offers a more comprehensive evaluation of the coagulation process and may facilitate the assessment of platelet-related (dys)function^27,28^. It is conceivable that CTAD exerts a greater influence on PRP-based TGA because of its inhibitory effects on platelet activation, although direct evidence supporting this hypothesis remains limited. A key consideration is that the potential benefits of CTAD in minimizing preanalytical platelet activation must be carefully balanced against the possibility of attenuating physiological platelet-dependent procoagulant activity during the assay.

Beyond methodological considerations, the practical limitations of CTAD tubes—including higher cost, limited availability, and restricted tube sizes—may constrain their use in clinical research. Tube size becomes particularly relevant in paediatric settings, where only very small blood volumes can often be obtained. While citrate tubes are available in a wide range of volumes suitable for neonates and infants, CTAD tubes are typically produced only in standard adult sizes. In paediatric research, blood conservation is particularly critical, as repeated sampling can quickly exceed safe limits. The lack of appropriately sized CTAD tubes therefore represents a significant obstacle to their use, despite their potential advantages in reducing preanalytical platelet activation. Although some studies have evaluated the feasibility of conducting coagulation tests from partially filled CTAD tubes^29,30^, this limitation frequently necessitates reliance on citrate tubes in practice even when CTAD might be preferable from a methodological standpoint. The present study was motivated in part by this challenge and aimed to evaluate whether citrate tubes may be considered an acceptable alternative when CTAD is impractical or unavailable.

Previous studies have reported divergent findings regarding differences between standard citrate and CTAD blood sampling methods. Lacroix et al. observed no significant differences in microparticle count, thrombin generation, or coagulation time between citrate and CTAD samples^31^. However, their research question and methodology differed from ours, and fewer experimental conditions were assessed. More recently, studies comparing CTAD and citrate in the context of heparin monitoring have yielded mixed results. In 2023, Gremillet et al. reported comparable stability for aPTT and anti-factor Xa activity^32^, whereas Lasne et al. observed an approximately 15% increase in anti-Xa activity in CTAD samples^33^. Taken together, and in line with our findings, these studies highlight the potential—yet inconsistent—influence of standard citrate *versus* CTAD blood sampling on coagulation assays across different methodologies and patient populations.

An alternative anticoagulant strategy to the use of either trisodium citrate alone or CTAD is the addition of corn trypsin inhibitor (CTI) to trisodium citrate^11,24^. CTI inhibits factor XIIa, thereby reducing contact activation during blood collection^34,35^. This might be particularly important when TGA is performed using low tissue factor concentrations^34,35^. However, as highlighted by Depasse et al., the routine use of CTI is limited by its restricted commercial availability, high cost, and the potential for variability associated with in-house preparation, which complicates interlaboratory comparisons^24^.

Since its introduction in the 1950s, TGA has undergone substantial methodological improvements, facilitating its increasing implementation in routine laboratory practice^36–38^. Nevertheless, international standardization remains limited. In 2020, the Scientific and Standardization Committee of the International Society of Thrombosis and Hemostasis emphasized that variability in reagents, calibration, reference ranges, and preanalytical handling continues to hamper comparability across institutions^11,39^. Our findings add to this discussion by identifying the choice of blood collection tube as a potentially relevant preanalytical factor influencing TGA results. Importantly, we demonstrate that the impact of standard citrate *versus* CTAD blood sampling depends on the specific TGA assay conditions, thereby adding further granularity to the existing evidence. This nuanced understanding may assist investigators in selecting citrate tubes as a pragmatic alternative when CTAD sampling is impractical or unavailable for particular research questions.

Several limitations of this study need to be considered. First, the experimental in vitro setup included a small cohort of healthy volunteers, limiting the generalizability of our findings, particularly to patients with haemostatic disorders, or those receiving anticoagulant therapy. Second, the use of a single analytical platform restricts possible extrapolation of the results to other TGA systems or reagent kits. Third, preanalytical conditions were tightly controlled, including immediate processing, standardized centrifugation, and storage at − 80 °C, which may not fully reflect clinical workflows. Even in research settings, substantial variation in the interval between blood collection and sample processing has been reported, with delays of up to 120 minutes^11^. This contrasts with current recommendations that samples for TGA should be processed within 1 hour^40^. Although CTAD may provide advantages by limiting the release of platelet-derived plasma factors during storage^10^, Rodgers et al. found that delays of up to 6 h between blood collection and centrifugation did not affect thrombin generation in citrate-anticoagulated samples^41^. Further studies are required to better define the impact of this preanalytical factor. Fourth, only citrate and CTAD tubes from a single manufacturer were evaluated, whereas differences in tube composition between manufacturers may further influence assay results. This is particularly relevant as the designation “3.2% trisodium citrate” encompasses citrate concentrations ranging from 0.105 mol/L to 0.109 mol/L, depending on the manufacturer. The potential impact of these differences was not evaluated in the present study. Finally, one participant was identified as an outlier and excluded from the primary analysis due to unexplained deviation, which may have resulted from sampling or measurement error, or from subject-specific factors.

In conclusion, thrombin generation assays performed on CTAD samples from healthy individuals yielded different results compared with citrate samples at low phospholipid concentrations. This finding identifies an additional factor influencing TGA outcomes that should be considered when interpreting and comparing scientific data. Given the advantages and limitations of CTAD sampling, the choice of tubes should be guided by the specific research question or clinical context, balancing practical feasibility with the potential impact of preanalytical variability on assay results.

## Supplementary Information

Below is the link to the electronic supplementary material.

Supplementary Material 1

## Data Availability

The datasets used and analysed during the current study are available from the corresponding author on rea-sonable request.
