# Supplementary material for: Impact of blood collection tubes on thrombin generation assay results: a comparison of citrate and CTAD
Source: Sci Rep. 2026 Jul 31;16:23718. doi: 10.1038/s41598-026-64702-6 (PMC13427730; doi:10.1038/s41598-026-64702-6)
Supplement: Supplementary file 1 — Supplementary Material 1 [file 41598_2026_64702_MOESM1_ESM.docx]

*Supplemental Figure 1*


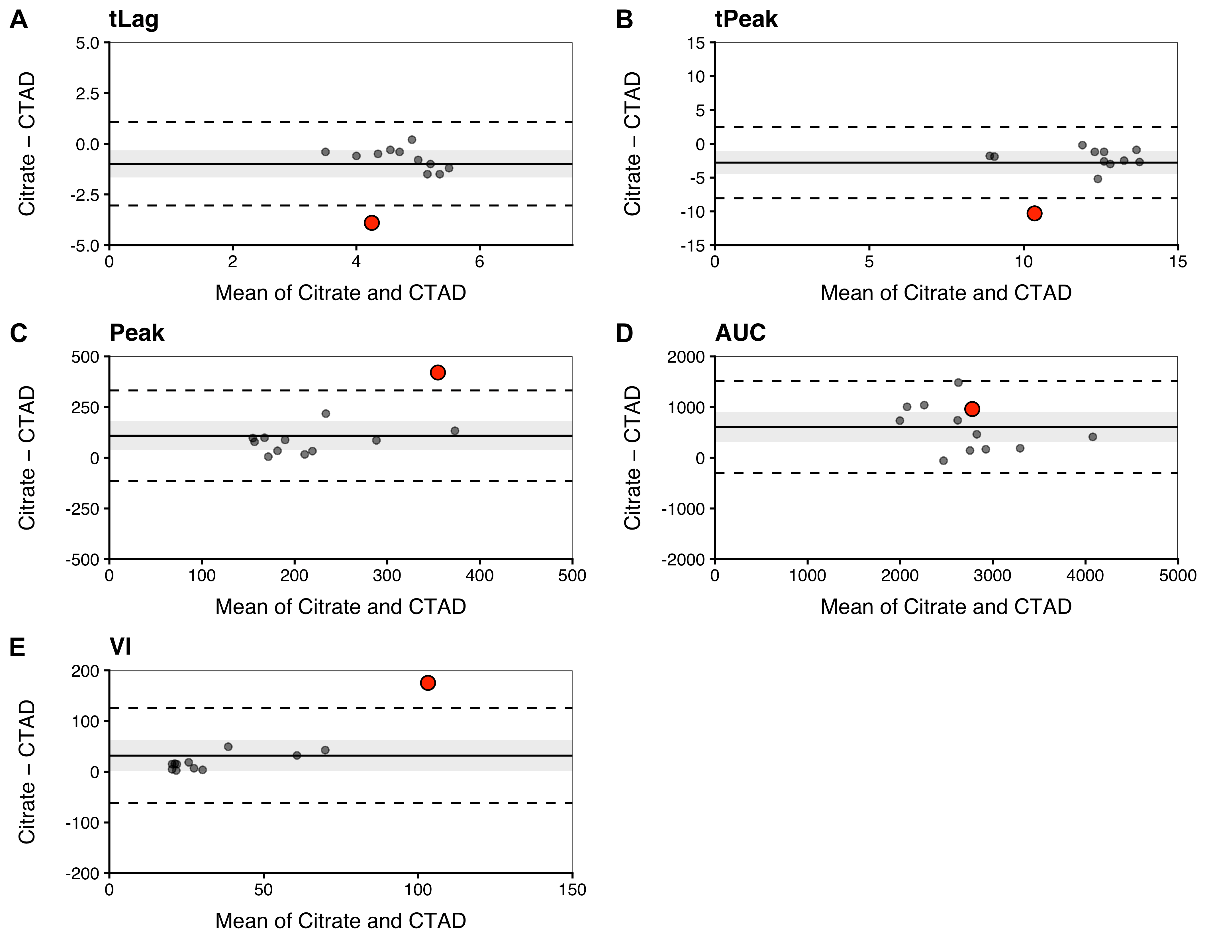


*Supplemental Figure 2*


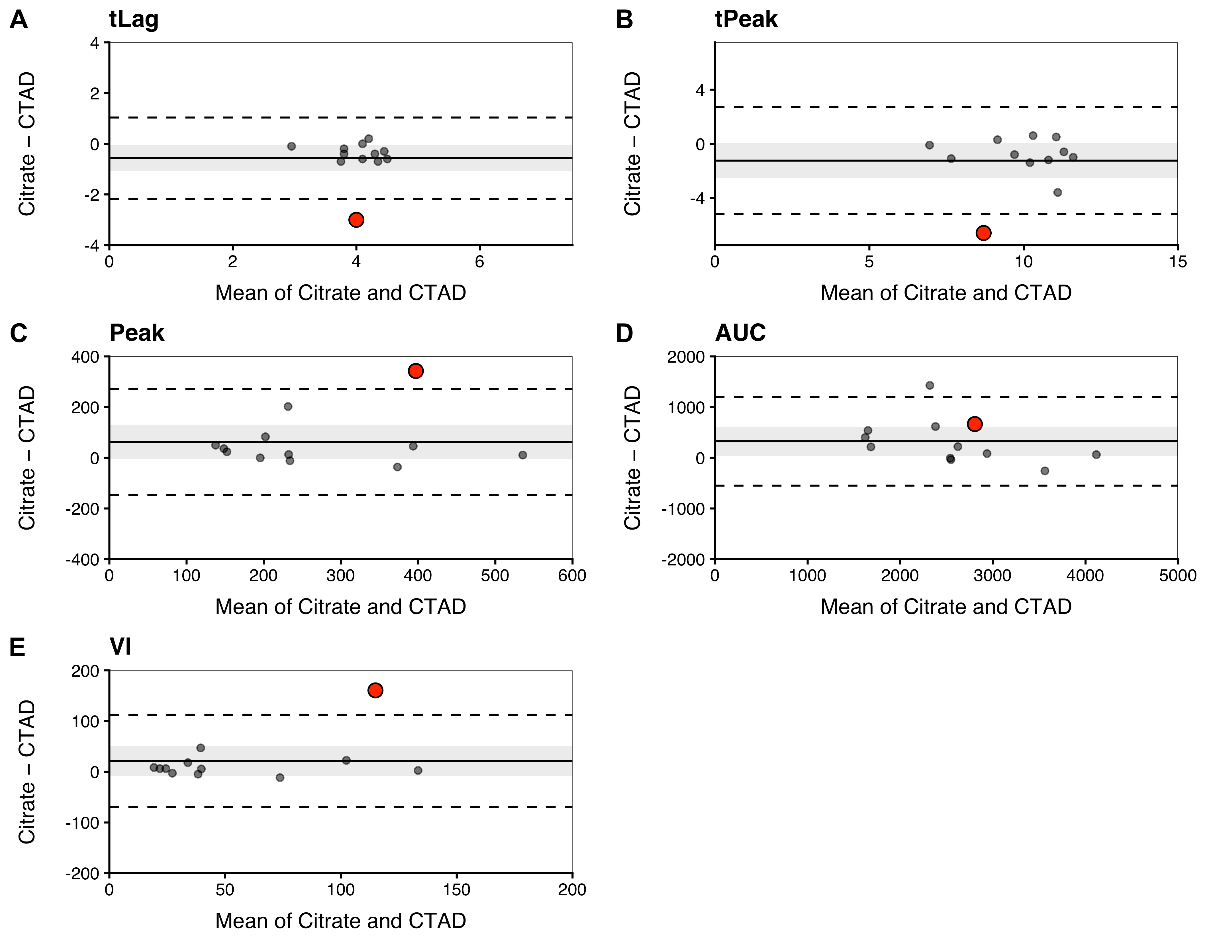


*Supplemental Figure 1* Differences between the two sampling groups in TGA with the RC low reagent in a Bland-Altman plot. *red* = statistical outlier

A) Bland-Altman plot for the tLag TGA parameter measured with the RC low reagent

B) Bland-Altman plot for the tPeak TGA parameter measured with the RC low reagent

C) Bland-Altman plot for the Peak TGA parameter measured with the RC low reagent

D) Bland-Altman plot for the AUC TGA parameter measured with the RC low reagent

E) Bland-Altman plot for the VI TGA parameter measured with the RC low reagent

*Supplemental Figure 2* Differences between the two groups in TGA with the RC high reagent in a Bland-Altmann plot. *red* = statistical outlier

A) Bland-Altman plot for the tLag TGA parameter measured with the RC high reagent

B) Bland-Altman plot for the tPeak TGA parameter measured with the RC high reagent

C) Bland-Altman plot for the Peak TGA parameter measured with the RC high reagent

D) Bland-Altman plot for the AUC TGA parameter measured with the RC high reagent

E) Bland-Altman plot for the VI TGA parameter measured with the RC high reagent
